# Spatial Omics in Gastrointestinal Oncology: Recent Advances, Therapeutic Insights, and Clinical Translation

**DOI:** 10.7150/jca.127381

**Published:** 2026-01-30

**Authors:** Lin Zhong, Qian Li, Ting Xiong, Shengzheng Lin, Kaining Wang, Guanying Li, Ye Yan, Jianhao Liu, Chuansong Xue

**Affiliations:** 1 Internal Medicine of Three, Sanya City Traditional Chinese Medicine Hospital, Sanya, China.; 2 Department of Oncology, Shanghai Children's Medical Center, Shanghai Jiao Tong University School of Medicine, National Health Committee Key Laboratory of Pediatric Hematology & Oncology, Shanghai, China.

**Keywords:** spatial omics, gastrointestinal cancer, tumor microenvironment, precision oncology, immunotherapy

## Abstract

Gastrointestinal (GI) cancers remain a leading cause of cancer-related morbidity and mortality worldwide, largely due to their molecular heterogeneity, complex tumor microenvironment (TME), and variable treatment responses. In recent years, the emergence of spatially resolved omics technologies—encompassing spatial transcriptomics, proteomics, metabolomics, and epigenomics—has revolutionized the ability to interrogate tumor architecture with unprecedented resolution. These methods enable precise mapping of cellular and molecular interactions within intact tissue contexts, thereby uncovering spatially defined niches that influence tumor progression, immune evasion, and therapeutic resistance. In GI malignancies such as colorectal, gastric, and esophageal cancers, spatial omics have provided critical insights into cancer-stromal-immune crosstalk, identified predictive biomarkers for immunotherapy and targeted agents, and guided the development of novel therapeutic strategies. This review synthesizes the latest advances in spatial omics applied to GI oncology over the past five years, with an emphasis on their integration into early diagnosis, treatment stratification, and real-time monitoring of therapeutic efficacy. We also discuss current challenges, including standardization, data integration, and clinical validation, as well as future directions for incorporating spatial profiling into routine oncology practice. By bridging the gap between bench discoveries and bedside applications, spatial omics hold transformative potential for achieving truly personalized treatment in gastrointestinal cancers.

## 1. Introduction

Gastrointestinal (GI) cancers, including gastric, colorectal, esophageal, pancreatic, and hepatobiliary malignancies, collectively represent one of the leading causes of cancer-related morbidity and mortality worldwide 1. Despite substantial progress in early detection, surgical techniques, and systemic therapies, the prognosis for advanced-stage GI cancers remains poor, with five-year survival rates often below 20% for metastatic disease 2. A major reason for this therapeutic gap lies in the profound spatial and molecular heterogeneity within tumors and their microenvironments, which drives treatment resistance, immune evasion, and metastatic spread 3.

Spatial technologies—particularly spatial transcriptomics, spatial proteomics, and high- dimensional imaging—have emerged as transformative tools in oncology over the past five years 4. Unlike bulk or single-cell sequencing methods, which disrupt the tissue context, spatial profiling preserves the architectural integrity of tumor specimens, enabling direct visualization and quantification of molecular features within their native microanatomical context 5. In GI oncology, this capability offers unprecedented opportunities to map tumor-stroma-immune cell interactions, dissect gradients of hypoxia or metabolic stress, and trace drug penetration patterns within heterogeneous tumor regions 6.

Recent studies have highlighted the translational value of spatial technologies for therapy optimization. For instance, spatial transcriptomic profiling has revealed immune-excluded phenotypes in gastric cancer that are resistant to immune checkpoint inhibitors, while also identifying microenvironmental niches that could be targeted by combination anti-angiogenic and immunotherapy regimens 7. Similarly, spatial proteomic mapping using imaging mass cytometry has demonstrated how chemotherapy reshapes the immune microenvironment in colorectal cancer, providing predictive signatures for therapeutic response 8. This review aims to synthesize recent advances in spatial technologies as applied to GI cancers, with a specific focus on their therapeutic relevance—from biomarker discovery to clinical trial integration—highlighting both scientific progress and remaining challenges.

## 2. Principles and Evolution of Spatial Technologies in Oncology

The development of spatial technologies in oncology reflects a paradigm shift from descriptive histopathology to quantitative, multi-omic, and high-resolution molecular cartography. Early methods, such as immunohistochemistry (IHC) and fluorescence in situ hybridization (FISH), enabled spatially resolved detection of a limited number of targets but were constrained by low multiplexing capacity and semi-quantitative analysis 9. Over the past decade, innovations in molecular capture chemistry, barcoding strategies, and imaging modalities have led to the emergence of powerful platforms capable of profiling thousands of transcripts or proteins simultaneously within intact tissue sections 10.

Spatial transcriptomics technologies, such as 10x Genomics Visium, NanoString GeoMx Digital Spatial Profiler, Slide-seqV2, and high-resolution methods like MERFISH (Multiplexed Error-Robust Fluorescence *In Situ* Hybridization), provide distinct trade-offs in spatial resolution, transcriptome coverage, and throughput 11. Complementary spatial proteomics platforms—including imaging mass cytometry (IMC), multiplexed ion beam imaging (MIBI), and cyclic immunofluorescence (CyCIF)—offer high-plex protein detection with subcellular resolution, enabling functional phenotyping of tumor and immune cell subsets within their native architecture 12. More recently, epigenomic spatial mapping techniques, such as spatial CUT&Tag and spatial ATAC-seq, have extended the analytical reach to chromatin accessibility and histone modification landscapes, further enriching the mechanistic understanding of tumor biology 13.

**Figure 1:**
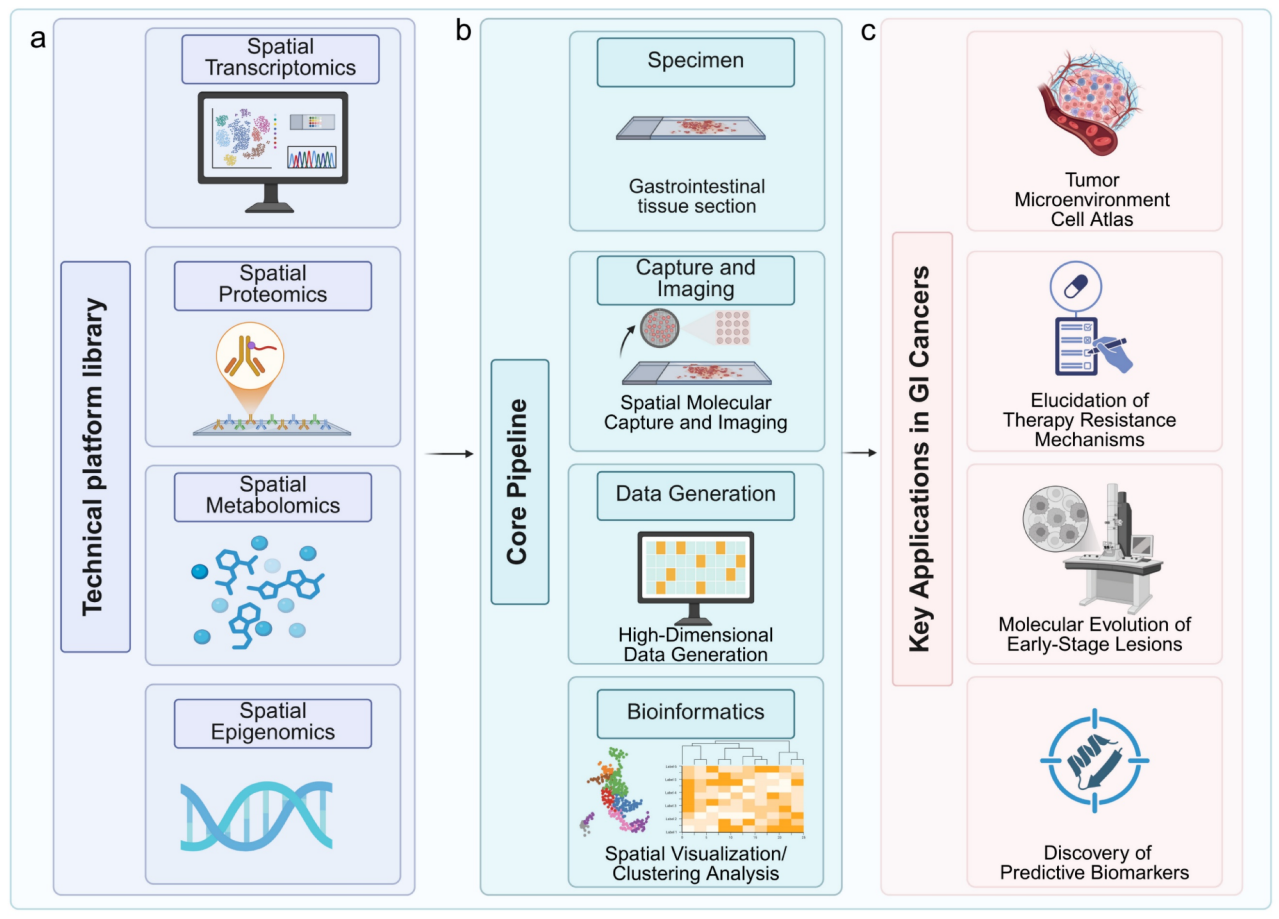
Workflow of spatially resolved multi-omics for therapeutic target identification and validation in gastrointestinal cancers. (a) The pipeline begins with gastrointestinal tissue sections subjected to spatial transcriptomics, proteomics, metabolomics, and epigenomics platforms. (b) Following molecular capture and imaging, high-dimensional data are generated and processed through bioinformatic analyses for spatial visualization and clustering. (c) Key applications include construction of tumor microenvironment atlases, elucidation of therapy resistance mechanisms, tracking molecular evolution in early-stage lesions, and discovery of predictive biomarkers. Image created with BioRender.com, with permission.

In GI cancer research, these technologies have been rapidly adopted to tackle questions previously inaccessible with conventional bulk or single-cell omics. For example, spatial transcriptomics has been used to identify spatially confined cancer stem cell niches in colorectal tumors that exhibit resistance to chemoradiotherapy 14, while spatial proteomics has characterized immune infiltration patterns in microsatellite instability-high (MSI-H) versus microsatellite stable (MSS) colorectal cancers, revealing prognostic immune signatures 15. The ability to integrate spatial transcriptomic, proteomic, and epigenomic layers within the same tissue sample has also advanced the field toward true spatial multi-omics, enabling more precise therapeutic hypothesis generation (Fig. 1a-c). Equipped with these foundational principles and evolving platforms, spatial omics is now being deployed to deconstruct the spatially organized complexity of the GI TME. This application is yielding transformative insights, particularly in elucidating how the precise spatial arrangement of tumor, immune, and stromal cells governs disease progression and modulates response to therapy.

## 3. Spatial Profiling in Gastrointestinal Tumor Microenvironment Analysis

The tumor microenvironment (TME) plays a decisive role in GI cancer progression and therapeutic outcomes. Spatial profiling has illuminated the complexity of the GI TME by mapping not only the cellular composition but also the spatial relationships that underpin functional states. In gastric cancer, Cousin et al. applied spatial transcriptomics to biopsies from patients treated with anti-PD-1/PD-L1 plus anti-angiogenic therapy, identifying microenvironmental regions enriched in fibroblast activation protein (FAP)-positive cancer-associated fibroblasts (CAFs) and M2-like macrophages that correlated with primary resistance to therapy 7. These resistant niches were spatially segregated from cytotoxic T-cell clusters, suggesting a physical and biochemical barrier to immune infiltration.

**Figure 2:**
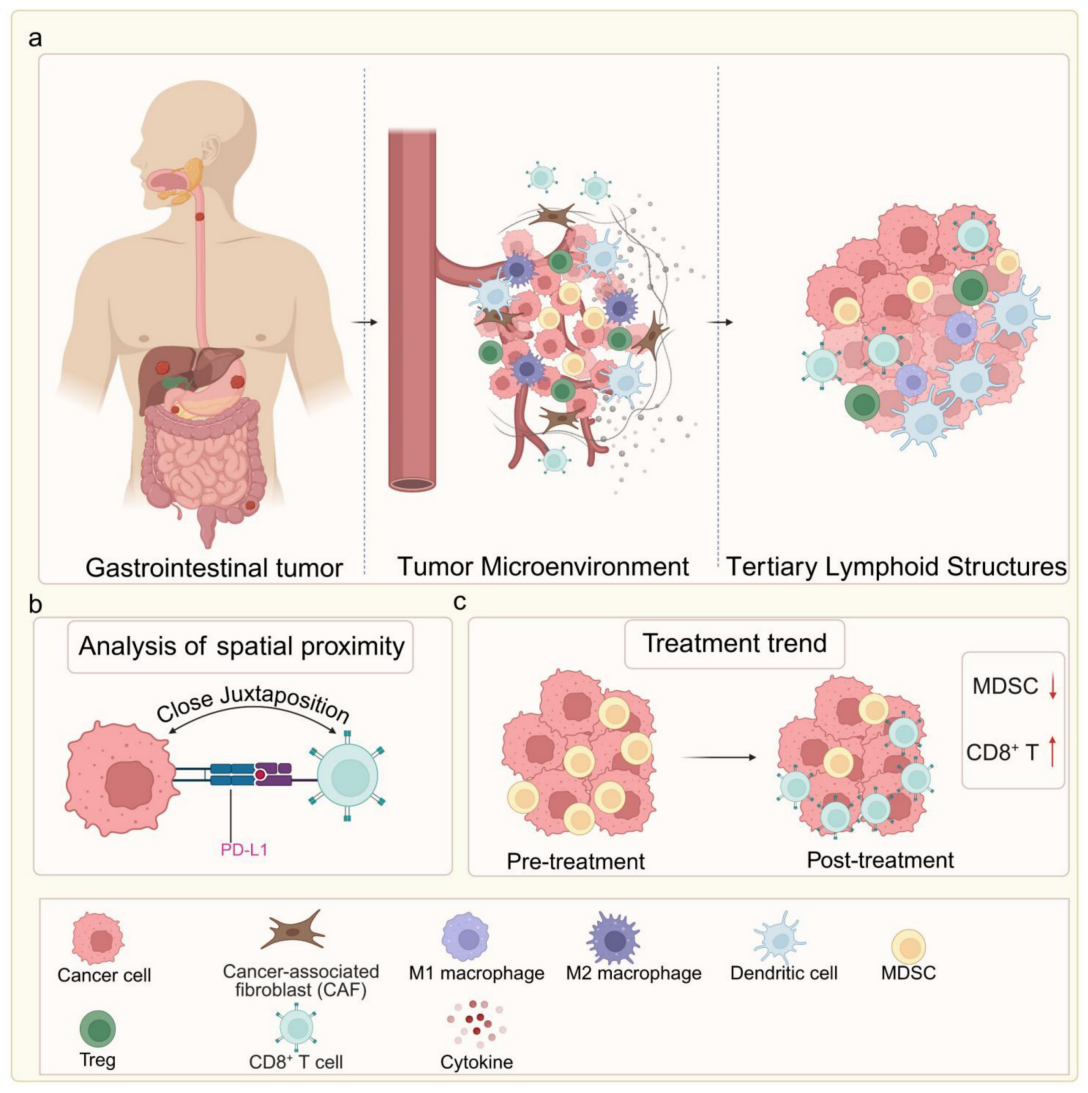
Spatial architecture of the GI tumor microenvironment and its association with therapeutic response. (a) Comparison of two key immune phenotypes: The immune-excluded phenotype features a CAF-rich barrier that physically blocks CD8⁺ T cell infiltration, leading to primary immunotherapy resistance. The immune-inflamed phenotype shows extensive intermingling of CD8⁺ T cells with tumor cells and presence of tertiary lymphoid structures (TLS), correlating with favorable treatment response. (b) High-plex spatial proteomics reveals that close spatial juxtaposition of PD-L1⁺ tumor cells and CD8⁺ T cells serves as a predictive biomarker for immunotherapy response. (c) Dynamic remodeling of the TME in response to therapy: non-responders maintain immunosuppressive niches rich in MDSCs, whereas responders exhibit expansion of activated, CD8⁺ T cell proximal to residual tumor cells. Image created with BioRender.com, with permission.

Similarly, in colorectal cancer, high-dimensional spatial proteomics has shown that the density and proximity of PD-L1⁺ tumor cells to CD8⁺ T cells can predict responsiveness to immune checkpoint blockade, with closer juxtaposition indicating better outcomes 16. Other spatial studies have identified tertiary lymphoid structures (TLS) within the TME as favorable prognostic indicators, particularly in MSI-H tumors, where their abundance correlates with durable immunotherapy responses 17. In hepatocellular carcinoma and pancreatic cancer, spatial multi-omics approaches have revealed zonated immune suppression, with specific perivascular niches harboring regulatory T cells and myeloid-derived suppressor cells, potentially explaining poor immunotherapy efficacy in these tumors 18 (Fig. 2a-c).

Beyond immune profiling, spatial mapping of drug distribution has important implications for therapeutic design. Imaging mass spectrometry and spatial metabolomics have been used to track chemotherapeutic agents and their metabolites within tumor sections, revealing uneven drug penetration that aligns with hypoxic or fibrotic regions 19. Li et al. employed air-flow-assisted desorption electrospray ionization mass spectrometry imaging (AFADESI-MSI) to spatially map the distribution of paclitaxel and its metabolites in xenografts derived from gastric cancer patients. The study revealed heterogeneous drug accumulation, characterized by lower concentrations in regions rich in fibroblasts and dense with extracellular matrix. This distribution spatially correlated with the upregulation of drug efflux transporters (ABCB1) and collagen-remodeling genes. By integrating these findings with spatial transcriptomics, researchers can identify molecular programs that drive drug resistance in areas with poor drug penetration, thereby informing targeted delivery strategies or combination therapies 20. Integrating these data with spatial transcriptomics can pinpoint molecular programs driving drug resistance in poorly penetrated areas, guiding targeted delivery strategies or combination therapies. Collectively, these findings underscore the utility of spatial profiling as both a discovery engine and a clinical decision-making tool in GI oncology, with the potential to refine patient stratification, inform rational drug combinations, and overcome resistance mechanisms. Beyond understanding established tumors, spatial technologies also offer promising tools for detecting early molecular and microenvironmental alterations, potentially transforming early diagnosis and risk stratification in GI cancers.

## 4. Applications in Early Detection and Diagnosis

Early detection of gastrointestinal (GI) cancers is critical for improving patient survival, yet current screening tools—such as endoscopy, imaging, and serum biomarkers—lack sufficient sensitivity and specificity for early-stage disease detection 21. Spatial omics technologies offer a novel avenue to identify early molecular and microenvironmental alterations in precancerous lesions, enabling more precise risk stratification. Spatial transcriptomics applied to gastric intestinal metaplasia and early dysplasia has revealed progressive immune and stromal remodeling preceding overt carcinoma, including expansion of myofibroblast clusters expressing WNT2B and NOTCH pathway ligands 22. In colorectal adenomas, spatial proteomic profiling using multiplex immunofluorescence has identified gradients of proliferative and immune-suppressive signaling emanating from dysplastic crypts, which correlate with subsequent malignant transformation 23.

A particularly promising application lies in integrating spatial data into AI-driven histopathology workflows. Deep learning models trained on multiplex spatial datasets can detect subtle tumor budding patterns and microenvironmental arrangements that are invisible to conventional hematoxylin and eosin (H&E) staining 24. Such approaches have been piloted in Barrett's esophagus and early gastric cancer, achieving area under the receiver operating characteristic curve (AUC) scores above 0.9 for high-risk lesion prediction. Moreover, spatial metabolomics has uncovered metabolic field effects—such as focal lactate accumulation and altered phospholipid distribution—that occur in morphologically normal mucosa adjacent to GI tumors, offering potential for non-invasive biomarker development 25. Together, these advances suggest that spatial technologies could transform early GI cancer detection from a morphology-driven to a molecular-architecture-driven paradigm.

The integration of artificial intelligence (AI), particularly deep learning and computer vision, has further amplified the diagnostic power of spatial omics. AI models trained on spatially resolved multi-omic datasets can identify subtle architectural patterns—such as tumor budding, immune cell spatial clustering, and stromal remodeling—that are imperceptible to conventional histopathological assessment. Moreover, AI-driven spatial data deconvolution enables the prediction of molecular subtypes and therapy responses directly from routine H&E-stained slides, bridging the gap between traditional pathology and molecular profiling. Such approaches not only enhance early detection accuracy but also pave the way for scalable, automated spatial biomarker discovery in digital pathology workflows. These diagnostic advances naturally extend into the therapeutic realm, where spatial mapping is providing novel insights into mechanisms of treatment response and resistance.

## 5. Therapeutic Insights from Spatial Mapping

Spatial profiling has elucidated key mechanisms of therapeutic resistance and response in GI cancers. In the immunotherapy domain, spatial transcriptomics has been instrumental in defining immune-excluded phenotypes, where dense collagen deposition and CAF-rich barriers spatially segregate cytotoxic lymphocytes from tumor nests 26. By contrast, immune-inflamed phenotypes, characterized by intermingling of tumor cells with activated CD8⁺ T cells and dendritic cells, have been linked to durable checkpoint inhibitor responses 27. These spatial signatures have been proposed as predictive biomarkers in ongoing clinical trials of pembrolizumab and nivolumab in gastric and colorectal cancers 28, 29.

Spatial proteomics has also provided insight into the effects of chemotherapy and targeted agents on the tumor microenvironment. In neoadjuvant-treated rectal cancer, multiplexed ion beam imaging revealed that responders exhibited post-treatment expansion of granzyme B⁺ T-cell clusters proximal to residual tumor cells, while non-responders retained immunosuppressive myeloid niches 15. Similarly, spatial metabolomic mapping of pancreatic ductal adenocarcinoma after stromal depletion therapy showed increased chemotherapeutic drug penetration in previously inaccessible tumor regions 29. These findings highlight the potential of spatial analysis not only to elucidate mechanisms of therapeutic action but also to identify combinatorial strategies—such as pairing stromal remodeling agents with immunotherapy or cytotoxic drugs—to overcome spatially defined resistance barriers. The therapeutic insights gained from spatial mapping are now being translated into clinical strategies, guiding precision oncology approaches that are both molecularly and spatially informed.

## 6. Guiding Precision Oncology in Gastrointestinal Cancers

The ultimate goal of spatial technology integration in GI oncology is to inform precision medicine strategies. Spatially resolved molecular data can refine patient stratification beyond genomic profiling alone, incorporating information about the location, context, and interaction of molecular features 30. For example, in colorectal cancer, integrating spatial transcriptomic and proteomic features into clinical decision algorithms improved prediction of response to adjuvant chemotherapy compared to TNM staging alone 31. In gastric cancer, combining HER2 spatial distribution maps with downstream signaling pathway activation patterns has been used to guide intratumoral injection of trastuzumab-loaded nanoparticles to HER2-enriched regions, enhancing therapeutic efficacy in preclinical models 32,33.

**Figure 3:**
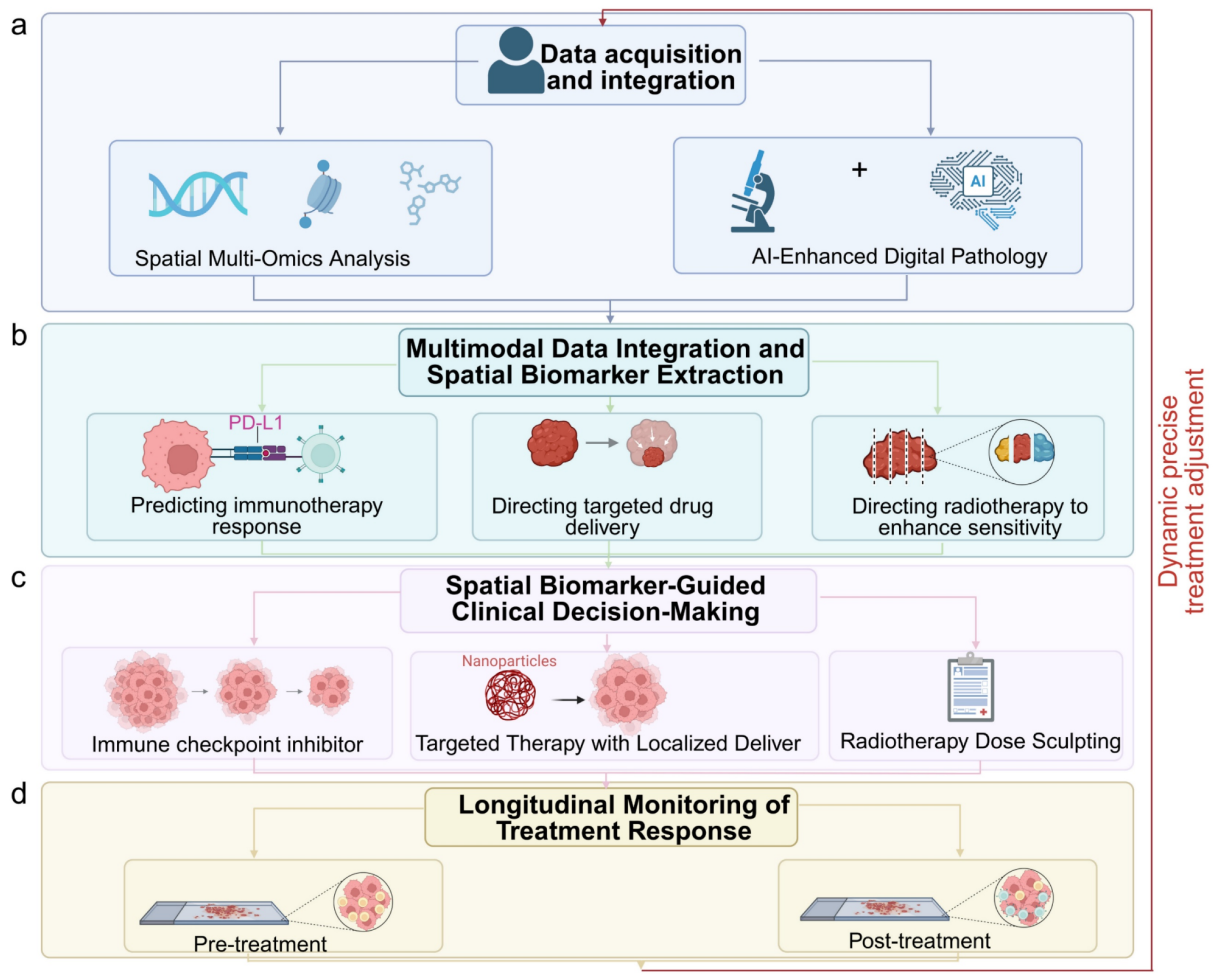
Integration of spatial multi-omics into clinical decision-making for precision oncology. (a) Data acquisition and integration from spatial multi-omics and AI-enhanced digital pathology. (b) Multimodal data integration for spatial biomarker extraction, such as PD-L1 expression patterns. (c) Spatial biomarker-guided clinical decisions, including localized drug delivery (e.g., nanoparticle-based targeting) and radiotherapy dose sculpting to resistant niches. (d) Longitudinal monitoring of treatment response through spatial profiling. Image created with BioRender.com, with permission.

Moreover, spatial data can inform surgical and radiotherapy planning. High-resolution mapping of hypoxic and therapy-resistant niches in esophageal cancer has enabled targeted dose escalation to spatially defined radioresistant zones 34. In hepatocellular carcinoma, spatial immune atlas construction from biopsy specimens has guided personalized combination regimens, selecting patients for anti-PD-1 plus anti-VEGF therapy based on the co-localization of exhausted T cells and VEGF-expressing endothelial cells 30-31,35. The ongoing integration of spatial omics into clinical trials, coupled with the emergence of standardized data formats and analytic pipelines, is accelerating the translation of these insights into practice 36. In the coming years, spatially informed precision oncology may shift the therapeutic paradigm for GI cancers toward strategies that are not only molecularly tailored but also anatomically and microenvironmentally optimized (Fig. 3a-d). The integration of multiple spatial omics layers—transcriptomic, proteomic, metabolomic, and epigenomic—further enhances our ability to comprehensively characterize tumors and uncover clinically actionable biomarkers.

## 7. Integrating Spatial Multi-Omics for Comprehensive Tumor Characterization

The rapid maturation of spatial multi-omics technologies, which integrate spatial transcriptomics, proteomics, metabolomics, and epigenomics, has enabled unprecedentedly comprehensive profiling of GI tumors 37, 38. By capturing multilayered molecular landscapes within intact tissue architecture, spatial multi-omics offers a unified view of tumor heterogeneity and its functional consequences. For instance, spatially resolved transcriptomics combined with MIBI has revealed colocalization patterns of immune checkpoint molecules and metabolic enzymes in CRC 39, 40, uncovering potential mechanisms of immune evasion linked to metabolic reprogramming.

Moreover, integration of spatial epigenomic data, such as histone modification mapping, with transcriptomic signatures has demonstrated how local chromatin states regulate gene expression gradients across the TME 41. This multi-dimensional approach has identified epigenetically controlled stromal niches in GC that foster immunosuppressive macrophage infiltration, with potential therapeutic implications 42. In HCC, spatial metabolomics using matrix-assisted laser desorption/ionization mass spectrometry imaging (MALDI-MSI) combined with proteogenomics has revealed discrete lipid-rich regions that correlate with resistance to tyrosine kinase inhibitors 43, 44, offering a spatially defined biomarker for treatment stratification.

These integrative pipelines not only illuminate tumor biology but also provide clinically relevant biomarkers. For example, a recent study integrating spatial RNA-seq with IMC in metastatic CRC successfully predicted patient response to anti-PD-1 therapy by quantifying spatially co-localized cytotoxic T cell-tumor cell interactions. Similarly, in GC, spatially informed co-analysis of the transcriptome and proteome has improved the prediction of HER2-targeted therapy outcomes beyond bulk tissue profiling. The clinical translation of spatial multi-omics will likely rely on harmonized data integration frameworks and machine learning algorithms capable of handling multi-scale, high-dimensional datasets.

The integrative power of spatial multi-omics thus offers an unprecedented lens for dissecting GI tumor biology. However, translating these high-dimensional, spatially resolved datasets into robust clinical tools necessitates overcoming substantial technical, analytical, and translational barriers. That said, realizing the full clinical translation of this paradigm requires resolving critical technical, analytical, and practical challenges, which will be the focus of our subsequent discussion.

## 8. Challenges, Limitations, and Future Perspectives

Despite its transformative potential, several challenges hinder the routine adoption of spatial technologies in GI cancer research and clinical care. First, technical limitations remain, including restricted spatial resolution for some platforms, limited throughput, and variable sensitivity across tissue types 10. For example, high-resolution platforms such as Slide-seqV2 can achieve single-cell resolution but often require complex sample preparation and may struggle with large biopsy specimens typical of clinical workflows 45. To address these issues, ongoing developments in microfluidics, combinatorial barcoding, and in situ amplification are improving both resolution and scalability. Additionally, the adoption of standardized tissue processing protocols and quality control metrics across laboratories will enhance reproducibility and comparability of spatial data in multi-center studies 46.

Second, the integration of multi-modal spatial datasets demands sophisticated computational infrastructure and standardized analytical pipelines. The lack of universally accepted data formats and quality control metrics complicates cross-study comparisons and meta-analyses. Furthermore, the high cost of spatial assays, along with limited access to specialized instrumentation in low-resource settings, exacerbates disparities in research and clinical application. Efforts such as the Spatial Omics Data Hub (SODH) initiative and open-source tools (e.g., Squidpy, Giotto) are emerging to promote data sharing and analytical harmonization. To improve accessibility, developing cost-effective, multiplexed detection methods and fostering public-private partnerships for instrument sharing in resource-limited regions could help democratize spatial profiling.

Biological interpretation also presents a bottleneck. While spatial maps can reveal complex cell-cell interaction networks, distinguishing causative from correlative spatial relationships often requires orthogonal validation through functional assays 47. Additionally, inter-patient variability in spatial architecture—shaped by tumor subtype, anatomical location, and treatment history—necessitates large-scale cohort studies to establish robust clinical biomarkers. Future studies should integrate spatial profiling with functional perturbation models, such as CRISPR-based screening in patient-derived organoids or spatial-CUT&Tag for epigenetic editing, to establish causal links. Moreover, collaborative consortia (e.g., Human Tumor Atlas Network) are essential to curate large, annotated spatial datasets that capture inter- and intra-tumor heterogeneity across diverse patient populations 48.

Looking forward, advances in in situ sequencing chemistry, high-plex protein detection, and computational modeling are expected to address many of these constraints. The integration of spatial data with longitudinal sampling, such as serial biopsies during neoadjuvant therapy, could provide real-time insights into treatment dynamics. Moreover, coupling spatial profiling with patient-derived organoids or explant models may bridge the gap between descriptive spatial maps and mechanistic understanding 49. Ultimately, the widespread clinical adoption of spatial technologies in GI oncology will depend on the convergence of improved assay accessibility, validated predictive biomarkers, and integration into precision oncology decision-making frameworks. To expedite the translation process, we propose the following measures: (i) the formation of interdisciplinary working groups tasked with defining clinically relevant spatial biomarkers; (ii) the integration of spatial endpoints into prospective clinical trials; and (iii) the creation of regulatory guidelines for the validation of spatial assays in diagnostic contexts 50.

## 9. Conclusion

Spatial omics signifies a transformative advancement in gastrointestinal oncology, surpassing conventional histopathological and bulk molecular analyses by offering multidimensional insights into tumor biology while preserving architectural integrity. This review emphasizes how spatially resolved transcriptomic, proteomic, metabolomic, and epigenomic platforms collaboratively elucidate the functional geography of gastrointestinal tumors, revealing immune-excluded niches, metabolic symbiosis, stromal barriers, and epigenetic gradients that influence disease progression and therapeutic resistance. The incorporation of spatial biomarkers into clinical decision-making—ranging from early risk stratification and immunotherapy prediction to guided drug delivery and radiotherapy optimization—is progressing from translational research toward early-phase clinical validation.

The comprehensive clinical adoption of spatial omics depends on addressing several enduring challenges: the standardization of assays and analytical pipelines, ensuring cost-effectiveness and accessibility across various healthcare settings, and the development of robust, prospectively validated spatial biomarkers through large-scale multicenter cohorts. Future advancements are expected to concentrate on: (i) real-time spatial monitoring of tumor evolution during therapy through serial biopsies or liquid biopsy-based spatial proxies; (ii) hybrid AI-spatial models that combine multiscale omic data with digital pathology to provide automated diagnostic and prognostic support; and (iii) functional validation of spatially derived targets utilizing patient-derived organoids and in vivo imaging models.

As spatial technologies advance and become increasingly integrated into standard oncological practice, they herald a new era of precision medicine that is both genetically informed and optimized for spatial and microenvironmental factors. By connecting molecular cartography with clinical applicability, spatial omics is set to transform therapeutic strategies and enhance survival outcomes for patients with gastrointestinal cancers.
